# Antimicrobial Resistance and Biofilm in Bacteria from Rehabilitated *Sapajus libidinosus*

**DOI:** 10.1007/s10393-026-01777-7

**Published:** 2026-04-06

**Authors:** Denny Parente de Sá Barreto Maia Leite, Gustavo de Oliveira Alves Pinto, Maria Eduarda Uchôa Cavalcanti Moreira da Silva, Valdir Vieira da Silva, Lucilene Martins Trindade Goncalves, Maria Clara Feitosa de Albuquerque, Rafaela Silva Santos, Luana Thamires Rapôso da Silva, Yuri Marinho Valença, Karolina Rosa Fernandes Beraldo, Maria Aparecida Juliano, Pollyanne Raysa Fernandes de Oliveira, José Givanildo da Silva, Rinaldo Aparecido Mota

**Affiliations:** 1 Graduate Program in Animal Biosciences, Federal Rural University of Pernambuco (UFRPE), Recife, Pernambuco Brazil; 2 Undergraduate student, Department of Veterinary Medicine, Federal Rural University of Pernambuco (UFRPE), Recife, Pernambuco Brazil; 3 Graduate Program in Veterinary Medicine, Federal Rural University of Pernambuco (UFRPE), Recife, Pernambuco Brazil; 4 Wildlife Screening and Rehabilitation Center (CETRAS-Tangara), Recife, Pernambuco Brazil; 5 Department of Biophysics, Paulista School of Medicine, Institute of Environmental, Chemical and Pharmaceutical Sciences, Federal University of São Paulo (UNIFESP), São Paulo, SP Brazil; 6 Department of Preventive Veterinary Medicine and Animal Production, School of Veterinary Medicine and Animal Science, Federal University of Bahia (UFBA), Salvador, Bahia Brazil

**Keywords:** microbial ecology, conservation, wildlife, antimicrobial resistance

## Abstract

Antimicrobial resistance (AMR) in natural environments and wildlife is an escalating threat to global health and biodiversity conservation. Neotropical primates of the genus *Sapajus* may act as reservoirs and ecological sentinels of resistant bacteria. The absence of systematic microbiological screening in wildlife rehabilitation centers, coupled with empirical antimicrobial use, can facilitate resistance spread in vulnerable ecosystems. This study characterized phenotypic and genotypic resistance profiles and biofilm-forming ability of *Staphylococcus* spp. and *Mammaliicoccus sciuri* isolated from *Sapajus libidinosus* undergoing rehabilitation in Northeastern Brazil. Rectal swabs were collected, and bacterial isolates identified by MALDI-TOF MS, followed by antimicrobial susceptibility testing, molecular detection of resistance genes, and biofilm assays. Nineteen isolates were recovered: 63.2% *Staphylococcus* spp. and 36.8% *Mammaliicoccus* spp. The predominant species were *M. sciuri* (36.8%) and *S. simiae* (31.6%). Rates of resistance to penicillin (63.2%) and tetracycline (57.9%) were the most frequent. The main resistance genes detected included *tetM* (36.8%), *tet(38)* (31.6%), *blaZ* (26.3%), *msrA* (26.3%), and *mecA* (5.3%). Perfect agreement existed between *mecA* presence and cefoxitin resistance (κ = 1.00; *p* < 0.01), with moderate agreement between *msrA* and non-susceptibility to erythromycin and clindamycin (j= 0.56; *p* = 0.0265). Biofilm production was mostly weak (94.7%), with moderate production in one isolate. Multidrug resistance occurred in 21.1% of isolates. This pioneering Brazilian study highlights wildlife rehabilitation centers as critical hotspots for AMR surveillance and contributes to understanding the ecological health and conservation of Neotropical primates.

## Introduction

Antimicrobial resistance (AMR) is an escalating global health threat that also extends to environmental and biodiversity conservation (Ahmed et al. 2024). Resistant microorganisms have been detected in natural environments and wildlife, revealing ongoing selective pressures even in remote areas. This broadens the complexity of the problem and highlights its ecological dimension (Radhouani et al. 2014; Bengtsson-Palme et al. 2023).

Wildlife species may act as ecological sentinels and reservoirs of antimicrobial resistance genes (ARGs), playing an active role in transmission networks between natural and anthropized environments (Arnold et al. 2016; Blanco-Peña et al. 2024). The interaction between wildlife, humans, and domestic animals in overlapping habitats facilitates this dynamic, especially in the case of highly adaptable microorganisms such as those from the genera *Staphylococcus* and *Mammaliicoccus*, which are widely distributed and known for their ability to acquire resistance traits (Radhouani et al. 2014; Baros Jorquera et al. 2021).

In this context, the rehabilitation and subsequent release of wild animals without proper microbiological screening poses a sanitary risk (Pérez Maldonado et al. 2025). Individuals exposed to empirical antimicrobial use during captivity may acquire multidrug-resistant strains and, once reintroduced into the wild, contribute to the dissemination of resistant bacteria and ARGs (Baros Jorquera et al. 2021; Pérez Maldonado et al. 2025). The lack of systematic microbiological surveillance protocols in Wildlife Screening and Rehabilitation Centers (CETRAS) undermines conservation and management efforts by overlooking the potential contamination of vulnerable ecosystems (Mitchell 2023).

Among the animals commonly admitted to these centers, Neotropical primates of the genus *Sapajus*, particularly *Sapajus libidinosus*, stand out due to their wide geographic distribution, exploratory behavior, and high ecological plasticity. These primates are frequently found in human-modified environments and have been reported as carriers of zoonotic bacteria, including *Staphylococcus* spp., in both free-ranging and captive individuals (Lima et al. 2023).

Considering this, the present study aimed to characterize the phenotypic and genotypic profiles of antimicrobial resistance and the biofilm-forming ability of *Staphylococcus* spp. and *Mammaliicoccus sciuri* isolates obtained from *S. libidinosus* individuals undergoing rehabilitation in a CETRAS facility in Northeastern Brazil. The findings contribute to strengthening integrated microbiological surveillance and promoting safer and more responsible wildlife conservation strategies.

## Material and Methods

### Ethical Authorization

This study was approved by the Animal Research Ethics Committee of the Federal Rural University of Pernambuco (CEUA-UFRPE, protocol no. 4094160421), the Biodiversity Authorization and Information System (SISBIO license no. 78418), and the National System for the Management of Genetic Heritage and Associated Traditional Knowledge (SisGen license no. AC42592).

### Study Site and Characterization of the Sampled Population

**Figure 1:**
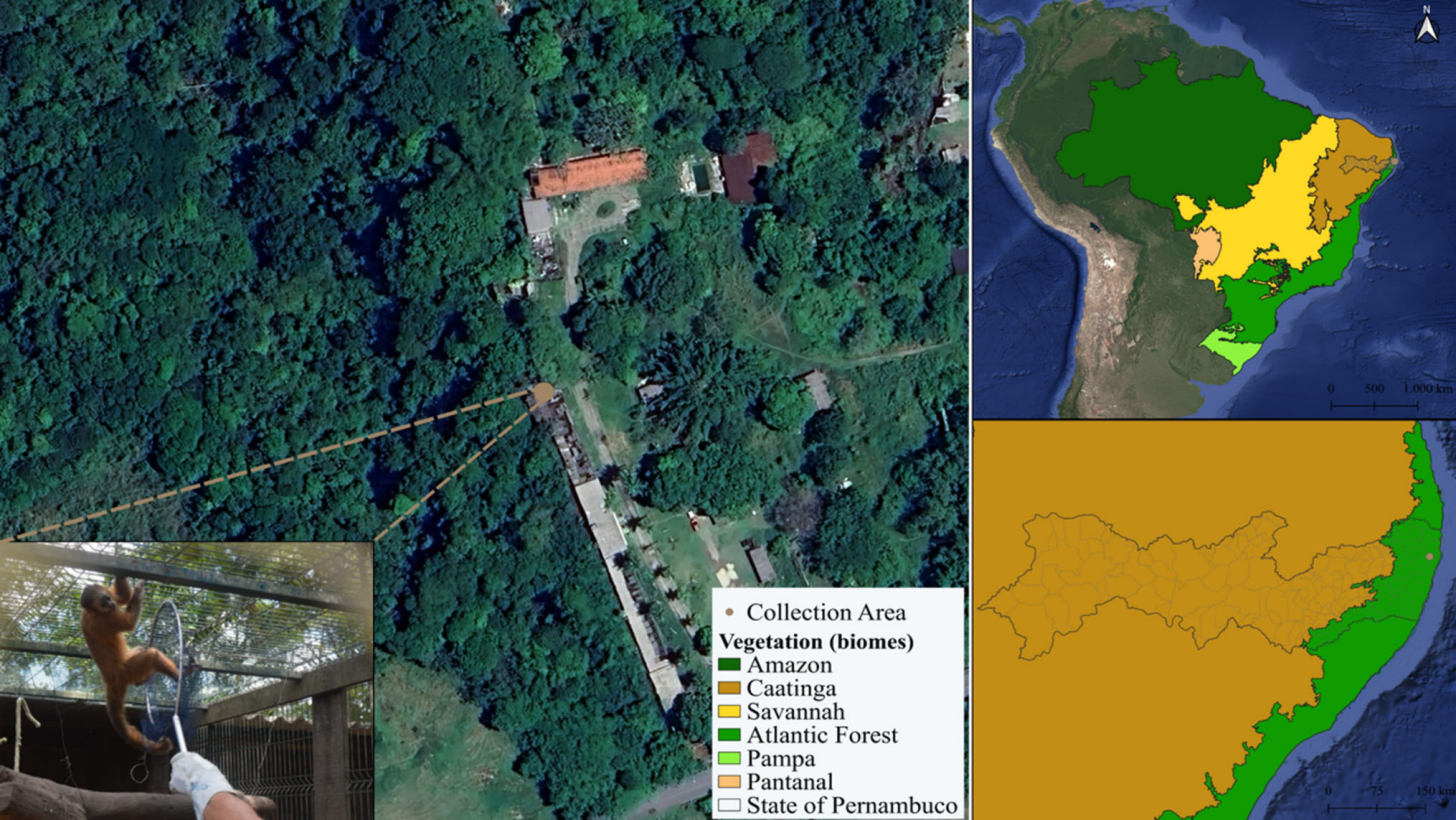
Location and rehabilitation flow of *Sapajus libidinosus* from captivity to release in Northeastern Brazil.

The present study was conducted at Wildlife Screening and Rehabilitation Center (CETRAS-Tangara), located in Recife, Pernambuco, northeastern Brazil, within the Atlantic Forest biome, and operated under the supervision of the state environmental agency (Fig. 1).

 The 20 *Sapajus libidinosus* individuals included in this study were initially housed in captivity at Parque Treze de Maio (Recife, Pernambuco). Although individual medical histories were incomplete, records indicated prolonged captivity under inadequate management conditions.

During their stay at Parque Treze de Maio, animals were kept under suboptimal environmental and nutritional management conditions. Environmental husbandry was characterized by high stocking density (multiple individuals housed in a single enclosure) and chronic exposure to auditory and visual stressors typical of a centrally located urban park, compounded by constant, unmonitored public visitation without effective control of proximity and interaction with the enclosure. Nutritional management was considered inadequate due to the lack of control over the recurrent provision of processed human foods by visitors over several years, with potential implications for nutritional status and gastrointestinal health.

All individuals were subsequently transferred to CETRAS Tangara for screening and rehabilitation (Fig. 1). One animal died shortly after admission (gastric ulcer perforation), and two were deemed unfit for release due to advanced age and chronic conditions (cataract and severe periodontitis). Thus, 17 individuals proceeded in the rehabilitation/release assessment process. Of these, all 17 individuals were ultimately released into a designated Wildlife Release Area (ASAS) in the semi-arid region of Pernambuco (Caatinga biome).

### Procedures for Capturing and Restraining the Animals

**Figure 2:**
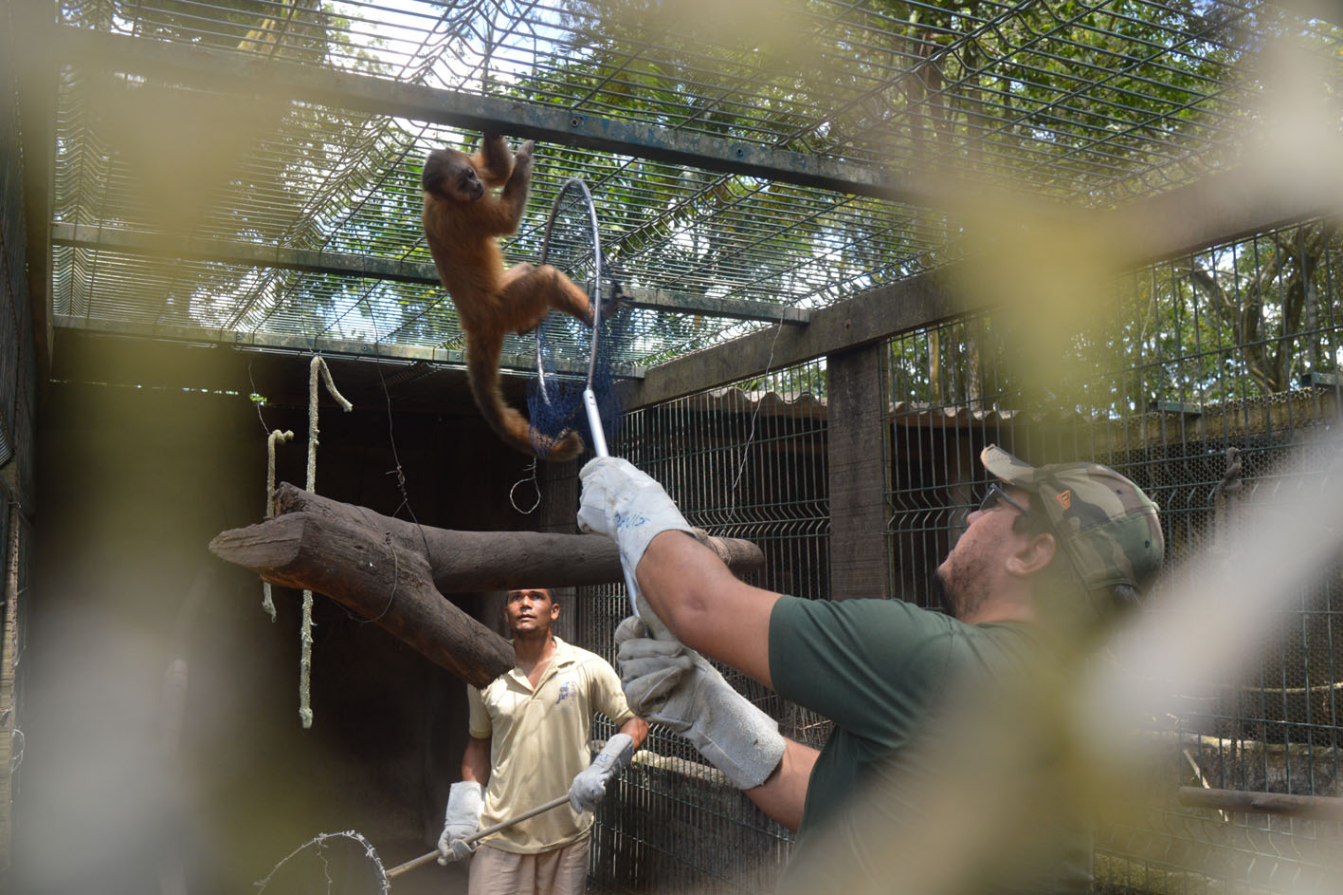
Handling of *Sapajus libidinosus* in a rehabilitation facility in Northeastern Brazil.

Both physical and chemical methods were used to restrain the animals. Initially, individuals were captured within the rehabilitation enclosures at CETRAS Tangara using individual physical restraint techniques, including the use of hand nets and leather gloves. After physical capture (Fig. 2), the animals were weighed to calculate the appropriate anesthetic dose for chemical restraint. Ketamine hydrochloride (5 mg/kg) and midazolam (0.08 mg/kg) were administered intramuscularly to induce hypnosis, sedation, and muscle relaxation (Verona and Pissinatti 2014). Once immobilized, each animal was placed in a transport crate and taken to the on-site veterinary clinic at CETRAS Tangara, where it underwent oxygen supplementation/oxygenation, stabilization, and microchipping before being subjected to physical examination and sample collection.

### Sample Collection

Initial physical restraint was used solely for the administration of sedative agents. Sample collection was performed during the physical examination while animals were under chemical restraint. Rectal swabs were placed in Stuart transport medium (Model 23010P, Absorve®, Jiangsu, China), properly labeled, and kept refrigerated in an insulated container with reusable ice packs until delivery to the Infectious Diseases Laboratory at the Federal Rural University of Pernambuco for microbiological and molecular processing.

### Bacterial Isolation and Identification

Rectal swab samples were inoculated onto Brain Heart Infusion (BHI) agar and Mannitol Salt Agar (MSA) and incubated at 37°C for 24–48 h. After incubation, plates were examined for bacterial growth, and presumptive colonies were subjected to Gram staining and catalase testing (Quinn et al. 2016).

Colonies displaying phenotypic characteristics consistent with *Staphylococcus* spp. and *Mammaliicoccus* spp. were subcultured on fresh MSA plates. For molecular analyses, bacterial biomass corresponding to one standard inoculation loop (approximately 10 µL) was collected from pure cultures for DNA extraction. The remaining culture was grown in BHI broth at 37°C for 24 h and cryopreserved at −80°C in BHI supplemented with 20% glycerol (Leite et al. 2023). Species-level identification was performed by matrix-assisted laser desorption/ionization time-of-flight mass spectrometry (MALDI-TOF MS), following the protocol described by Clark et al. (2013).

### Antimicrobial Susceptibility Testing

Phenotypic antimicrobial resistance was evaluated using the disk diffusion method, adjusted to 0.5 McFarland turbidity scale, following the guidelines of the Clinical and Laboratory Standards Institute (CLSI) and the European Committee on Antimicrobial Susceptibility Testing (EUCAST). Tests were conducted on Mueller–Hinton agar using bacterial suspensions standardized to 0.5 McFarland. *Staphylococcus aureus* ATCC 25923 was included as quality control strain (Matuschek et al. 2014; EUCAST 2023; CLSI 2024).

The antimicrobials tested were selected based on their clinical relevance for staphylococcal infections in veterinary and public health contexts, as well as their importance in antimicrobial resistance surveillance programs. The antibiotic panel included penicillin (PEN, 10 IU), cefoxitin (CFO, 30 µg), ciprofloxacin (CIP, 5 µg), tetracycline (TET, 30 µg), erythromycin (ERI, 15 µg), and clindamycin (CLI, 2 µg). Interpretation of inhibition zones followed CLSI VET01S (7th edition) and EUCAST guidelines (CLSI 2024; EUCAST 2023). For *Mammaliicoccus sciuri*, interpretive criteria for coagulase-negative staphylococci were applied due to the absence of species-specific breakpoints (Leite et al. 2025).

Multidrug resistance (MDR) was defined as non-susceptibility to at least one agent in three or more antimicrobial classes, according to Magiorakos et al. (2012) and Sweeney et al. (2019).

### DNA Extraction and Genotypic Profiling of Antimicrobial Resistance

Genomic DNA was extracted from bacterial colonies using the thermal lysis method described by Fan et al. (1995). Following extraction, DNA concentration and purity were evaluated by spectrophotometry, with absorbance reading at 260 nm (Brakstad et al. 1992), using a Thermo Fisher Scientific spectrophotometer (Massachusetts, USA). Subsequently, conventional PCR assays were performed to detect antimicrobial resistance genes (Table 1), grouped according to antimicrobial class and the resistance mechanism involved. For β-lactams, blaZ (β-lactamase production) and mecA/mecC (target alteration via PBP2a/PBP2c) were investigated. For fluoroquinolones, norA and norC, associated with efflux pumps, were evaluated. Resistance to macrolides and streptogramin B was investigated by msrA (ABC-F/efflux-associated mechanism), whereas genes associated with the MLS_B resistance mechanism were investigated by ermA, ermB, and ermC (23S rRNA methylation). For tetracyclines, genes representing distinct mechanisms were investigated: tetM (ribosomal protection) and tetL and tet(38) (efflux pumps associated with reduced susceptibility to tetracyclines) (Table 1).

**Table 1:** Resistance Genes Investigated, Primer Sequences, and Respective References.

Resistance mechanism (category)	Gene	Antibiotic class(es)	Forward primer (5′–3′)	Reverse primer (5′–3′)	References
Enzymatic inactivation	*blaZ*	β-lactams (mainly penicillins)	AAGAGATTTGCCTATGCTTC	GGCAATATGATCAAGATAC	Sawant et al. (2009)
Target modification (PBP alteration)	*mecA*	β-lactams (methicillin/oxacillin; screened with cefoxitin)	TGGTATGTGGAAGTTAGATTGGGAT	CTAATCTCATATGTGTTCCTGTATTGGC	Nakagawa et al. (2005)
	*mecC*	β-lactams	CATTAAAATCAGAGCGAGGC	TGGCTGAACCCATTTTTGAT	Paterson et al. (2012)
Efflux pumps	*norA*	Fluoroquinolones	TGCAATTTCATATGATCAATCCC	AGATTGCAATTCATGCTAAATATT	Truong-Bolduc et al. (2003)
	*norC*	Fluoroquinolones	AAATGGTTCTTCTAAGCGACCAA	ATAAATACCTGAAGCAACGCCACC	Truong-Bolduc et al. (2006)
	*tet(38)*	Tetracyclines	TTCAGTTTGGTTATAGACAA	CGTAGAAATAAATCCACCTG	Truong-Bolduc et al. (2005)
	*tetL*	Tetracyclines	TCGTTAGCGTGCTGTCATTC	GTATCCCACCAATGTAGCCG	McMurry et al. (1987); Ng et al. (2001)
Ribosomal protection	*tetM*	Tetracyclines	GTGGACAAAAGGTACAACGAG	CGGTAAAGTTCGTCACACAC	Ng et al. (2001)
Ribosomal target modification (MLS_B phenotype)	*ermA*	Macrolides, lincosamides, streptogramin B	GCGGTAAACCCCTCTGAG	GCCTGTCGGAATTGG	Werckenthin (2000)
	*ermB*	Macrolides, lincosamides, streptogramin B	GGAACATCTGTGGTATGGCG	CATTTAACGACGAAACTGGC	Jensen; Frimodt-Møller; Aarestrup (1999)
	*ermC*	Macrolides, lincosamides, streptogramin B	CAAACCCGTATTCCACGATT	ATCTTTGAAATCGGCTCAGG	
Macrolide/streptogramin B resistance (non-MLS_B)	*msrA*	Macrolides and streptogramin B	ATCATGTGATGTAAACAAAAT	GCAAATGGTGTAGGTAAGACAACT	Wondrack et al. (1996)

### Phenotypic Evaluation of Biofilm Production

Biofilm formation was assessed using the microtiter plate method, as described by Pratt and Kolter (1998) and Stepanovic et al. (2000). Isolates were cultured on tryptic soy agar (TSA) and subsequently inoculated into tryptic soy broth (TSB) supplemented with 0.1% glucose. After incubation and standardization to 0.5 McFarland, biofilm formation was quantified by crystal violet staining and optical density measurement at 620 nm. *Escherichia coli* ATCC 35218 and sterile medium were used as positive and negative controls, respectively. Biofilm production was classified according to established OD-based criteria (Stepanovic et al. 2000).

### Statistical Analysis

Data were organized in spreadsheets using the Python programming language in the Google Colab® environment. Descriptive analyses included absolute and relative frequencies to characterize bacterial isolates, antimicrobial resistance profiles, resistance gene distribution, and biofilm production categories.

 Agreement between phenotypic antimicrobial resistance (disk diffusion results) and genotypic resistance profiles (presence of corresponding resistance genes) was assessed using the Cohen’s Kappa coefficient (κ). Kappa values were interpreted according to Landis and Koch (1977). The association between the number of resistance genes detected per isolate and the degree of biofilm production was evaluated using Spearman’s rank correlation coefficient (ρ), given the ordinal nature and non-normal distribution of the data. Statistical significance was set at 5% (*p* < 0.05) (Zar 2010).

## Results

A total of 19 bacterial isolates were recovered, of which 12/19 (63.2%) belonged to the genus *Staphylococcus* and 7/19 (36.8%) to the genus *Mammaliicoccus*. The most frequently identified species was *Mammaliicoccus sciuri* (7/19; 36.8%), followed by *Staphylococcus simiae* (6/19; 31.6%), *S. xylosus* (2/19; 10.5%), and *S. aureus*, *S. cohnii*, *S. haemolyticus*, and *S. pasteuri* (each 1/19; 5.3%).

**Figure 3:**
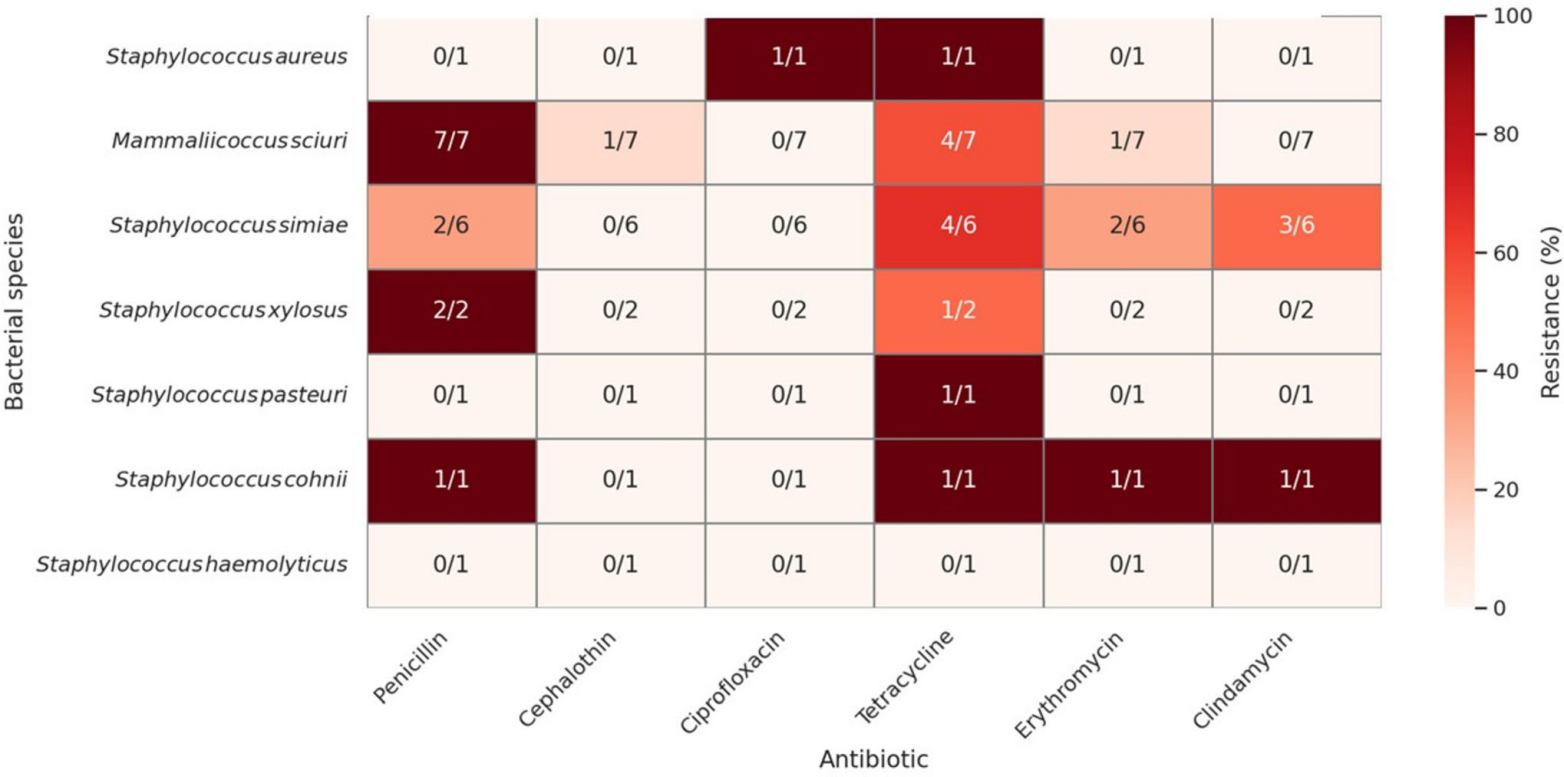
Heatmap showing the percentage of phenotypic resistance by bacterial species and tested antibiotics.

Regarding phenotypic antimicrobial resistance, 12/19 (63.2%) isolates were resistant to penicillin and 11/19 (57.9%) to tetracycline. Resistance to erythromycin and clindamycin was detected in each of the 4/19 (21.1%), while resistance to ciprofloxacin and cefoxitin was observed in 2/19 (10.5%) and 1/19 (5.3%) isolates, respectively. The distribution of resistance patterns by bacterial species is shown in Figure 3.

**Figure 4:**
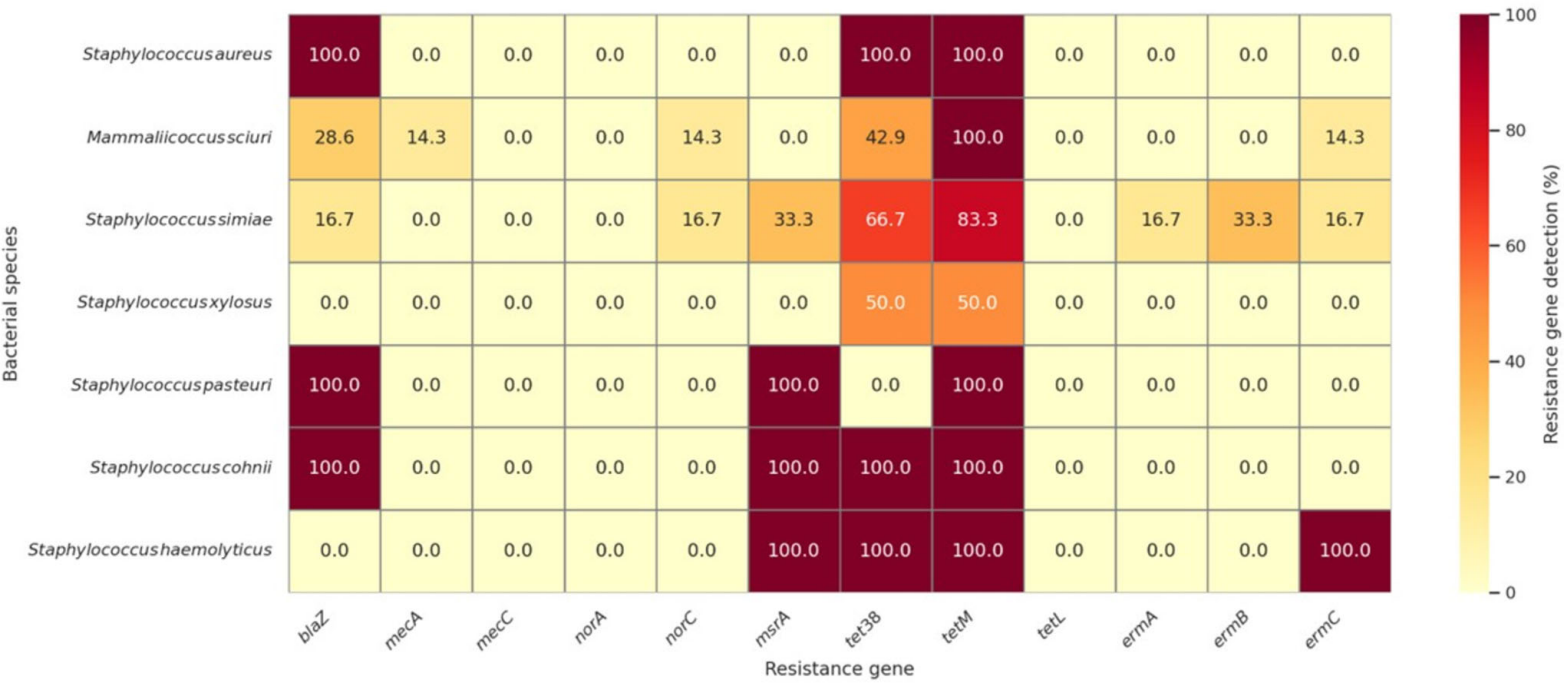
Heatmap of antimicrobial resistance gene detection by species, expressed as percentages.

Genotypic analysis showed that the most frequently detected resistance genes were *tetM* (7/19; 36.8%), *tet(38)* (6/19; 31.6%), *blaZ* (5/19; 26.3%), and *msrA* (5/19; 26.3%). The *mecA* gene was identified in 1/19 (5.3%) isolate. The distribution of resistance genes by species is presented in Figure 4.

**Figure 5:**
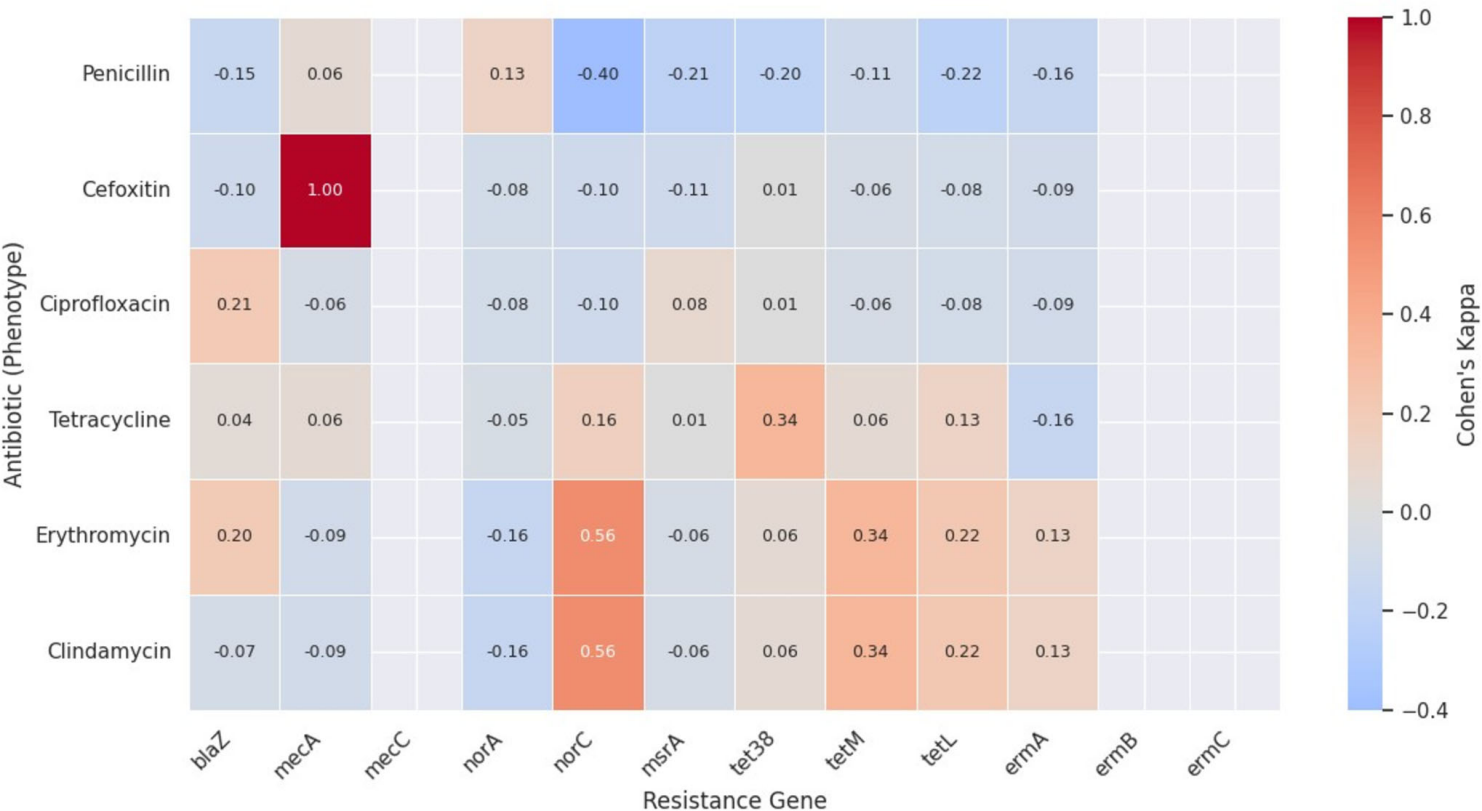
Heatmap of Cohen’s Kappa coefficient between phenotypic resistance profiles and corresponding resistance gene detection.

The concordance between phenotypic resistance and detection of resistance genes is shown in Figure 5. Perfect concordance was observed between cefoxitin resistance and the presence of *mecA* (κ = 1.00; *p* < 0.01). For macrolides, there was moderate concordance between *msrA* detection and non-susceptibility to erythromycin (κ = 0.56; *p* = 0.0265). Although Figure 5 also indicates moderate concordance between *msrA* and non-susceptibility to clindamycin (κ = 0.56), this finding should be interpreted with caution, as *msrA* does not confer resistance to lincosamides. Furthermore, the MLS_B induction test (D-test) was not performed, and other determinants of clindamycin resistance were not investigated. For most other gene-antimicrobial combinations, low or no concordance was observed, suggesting dissociation between genotype and phenotype in some of the isolates.

Regarding biofilm formation, 18/19 (94.7%) isolates were classified as weak biofilm producers, 1/19 (5.3%) as a moderate producer, and none as strong producers. The isolate classified as a moderate biofilm producer exhibited simultaneous resistance to penicillin, cefoxitin, and tetracycline. Among weak biofilm producers, resistance was most frequently observed to penicillin and tetracycline (11/18; 61.1%), followed by erythromycin and clindamycin (4/18; 22.2%). Resistance to ciprofloxacin (1/18; 5.6%) and cefoxitin (0/18; 0%) was rare.

The correlation between biofilm production and phenotypic antimicrobial resistance was weak. The highest Spearman correlation coefficient was observed for cefoxitin (ρ = 1.00), while penicillin and tetracycline showed slight positive correlations (ρ = 0.18). Negative correlations were observed for erythromycin, clindamycin, and ciprofloxacin.

Four out of 19 isolates (21.1%) were classified as multidrug-resistant (MDR). The *Staphylococcus cohnii* isolate exhibited resistance to penicillin, tetracycline, erythromycin, and clindamycin. Among the three MDR *Staphylococcus simiae* isolates, two were resistant to tetracycline, erythromycin, and clindamycin, while one showed resistance to penicillin, tetracycline, erythromycin, and clindamycin. A summary of species identification, phenotypic resistance, resistance genes, and biofilm-forming capacity is provided in Table 2.

**Table 2:** Phenotypic and Genotypic Resistance Profiles and Biofilm Production Capacity of Bacterial Isolates Recovered from *Sapajus libidinosus*.

ID	Species	Phenotypic resistance profile	Genotypic profile	Biofilm
MP3	*M. sciuri*	PEN, TET	*tet(38), tetM*	Weak
MP5.1	*M. sciuri*	PEN, TET	*norC, tet(38), tetM*	Weak
MP10	*M. sciuri*	PEN, ERI	*blaZ, tetM*	Weak
MP11	*M. sciuri*	PEN	*tet(38), tetM*	Weak
MP12	*M. sciuri*	PEN	*tetM, ermC*	Weak
MP18	*M. sciuri*	PEN, TET	*blaZ, tetM*	Weak
MP19	*M. sciuri*	PEN, CFO, TET	*mecA, tetM*	Moderate
MP1	*S. aureus*	CIP, TET	*blaZ, tet(38), tetM*	Weak
MP9	*S. cohnii*	PEN, TET, ERI, CLI	*blaZ, msrA, tet(38), tetM*	Weak
MP15.2	*S. haemolyticus*	–	*msrA, tet(38), tetM, ermC*	Weak
MP7	*S. pasteuri*	TET	*blaZ, msrA, tetM*	Weak
MP5.2	*S. simiae*	TET	*tet(38), tetM, ermB*	Weak
MP8	*S. simiae*	PEN	*norC, tet(38)*	Weak
MP14	*S. simiae*	TET, ERI, CLI	*msrA, tetM, ermA, ermC*	Weak
MP15.1	*S. simiae*	TET, ERI, CLI	*msrA, tet(38), tetM, ermB*	Weak
MP16	*S. simiae*	PEN, TET, CLI	*tetM*	Weak
MP17	*S. simiae*	–	*blaZ, tet(38), tetM*	Weak
MP6	*S. xylosus*	PEN, TET	*tet(38), tetM*	Weak
MP20	*S. xylosus*	PEN	–	Weak

PEN = Penicillin. TET = Tetracycline. ERI = Erythromycin. CLI = Clindamycin. CIP = Ciprofloxacin. CFO = Cefoxitin

During the rehabilitation period at CETRAS Tangara, some animals were subjected to empirical antimicrobial treatments as part of routine clinical management, primarily for traumatic skin and soft tissue injuries. The antimicrobials available and routinely used at the facility included systemic enrofloxacin and topical formulations of gentamicin and oxytetracycline. However, due to limited access to individual clinical records, it was not possible to determine which specific animals received antimicrobial treatment or to establish temporal associations between antimicrobial exposure and bacterial isolation. No information regarding antimicrobial use during the animals’ previous stay at Parque Treze de Maio was available.

Regarding waste management, fecal material and other organic residues from animal enclosures were routinely removed during cleaning procedures and disposed of in a rudimentary on-site composting area. This disposal site lacked physical barriers and access control, allowing contact with local synanthropic fauna. Such practices may represent a potential route for environmental dissemination of resistant bacteria, warranting consideration from a One Health perspective.

## Discussion

AMR in microorganisms associated with wildlife represents a One Health concern, particularly at interfaces where animals, humans, and the environment are closely interconnected. Wildlife screening and rehabilitation centers concentrate individuals from diverse origins, often requiring clinical interventions and shared housing conditions, which may facilitate microbial exchange. In this context, the present study provides culture-based data on *Staphylococcus* spp. and *Mammaliicoccus sciuri* recovered from rectal swabs of *Sapajus libidinosus* undergoing rehabilitation, integrating phenotypic antimicrobial susceptibility testing with targeted detection of resistance genes and assessment of biofilm formation.

The predominance of *M. sciuri* is consistent with previous findings reporting its presence in the cutaneous microbiota of primates (Wildsmith et al. 2025), and its detection in rectal mucosa expands current knowledge of its anatomical distribution. Although often described as part of the commensal microbiota, species of *Mammaliicoccus* have been reported, at animal–environment interfaces, as opportunistic microorganisms and carriers of resistance determinants (Dhaouadi et al. 2022; Moura et al. 2023). Isolates of this genus have been associated with resistance determinants to β-lactams and tetracyclines and may contribute to the broader ecological pool of AMR genes (Abdullahi et al. 2021; Moura et al. 2023).

Reports from animal production systems and natural environments also describe high genetic diversity and frequent detection of resistance determinants in *M. sciuri*, reinforcing its epidemiological relevance beyond a single host niche (Boonchuay et al. 2023).

In our isolates, phenotypic resistance was most frequently observed to penicillin and tetracycline, and the most detected genes included *blaZ* and tetracycline resistance determinants (*tetM* and *tet(38)*). These findings are consistent with previous reports, including studies conducted in captive primates in Brazil (García et al. 2020; Silva et al. 2019, 2020; Abdullahi et al. 2021; Ramos et al. 2022). However, our data does not allow source attribution; therefore, we avoid inferring whether the detected AMR determinants originated within the rehabilitation center, during the animals’ prior captivity, or from other exposures.

During the rehabilitation period at CETRAS Tangara, empirical antimicrobial treatments may be used as part of routine clinical management, particularly for traumatic skin and soft tissue injuries. Systemic enrofloxacin and topical formulations of gentamicin and oxytetracycline are available and routinely used; however, due to restricted access to individual clinical records, it was not possible to associate antimicrobial exposure with specific animals or to establish temporal relationships with bacterial isolation.

Studies conducted in captive primate colonies have demonstrated that antimicrobial pressure and clinical–veterinary procedures can shape colonization patterns, and that rational antimicrobial use programs may reduce colonization by resistant organisms over time (Kim et al. 2017; Breed 2023). In this sense, our findings reinforce the rationale for implementing structured antimicrobial stewardship programs in rehabilitation centers, while maintaining caution regarding causal interpretations.

The microorganisms detected also warrant consideration from both animal and human health perspectives. *Staphylococcus aureus* is a well-recognized opportunistic pathogen with zoonotic relevance, while several coagulase-negative staphylococci (*S. haemolyticus*, *S. cohnii*) and *Mammaliicoccus* species may act as opportunistic pathogens, particularly in compromised hosts or in the presence of wounds and invasive procedures (Tong et al. 2015; Rossi et al. 2020). These taxa may also function may also function as reservoirs of resistance determinants, with potential occupational implications in settings involving frequent animal handling (Hanley et al. 2015; Soge et al. 2016). Thus, even when isolates are obtained from colonization sites, documenting their resistance profiles contributes to risk assessment in clinical management and biosafety.

Genotype–phenotype concordance was limited for most gene–drug pairs, which is expected when conventional targeted PCR panels are used instead of comprehensive genomic approaches (Price et al. 2013; Palmer et al. 2018). Discordance may result from resistance mechanisms not included in the genetic panel, regulatory differences affecting gene expression, chromosomal mutations, or interpretative limitations when clinical breakpoints are extrapolated to less-studied taxa (Depardieu et al. 2007; Hadadi et al. 2018; Costa et al. 2019).

Evidence from broader comparisons between genotype and phenotype also indicates that molecular predictions do not perfectly match phenotypic susceptibility in all cases, particularly when molecular approaches do not capture the entire resistome (Stoesser et al. 2013; Ellington et al. 2017). Accordingly, our results support the continued use of standardized phenotypic susceptibility testing as the primary basis for interpretation, complemented by targeted molecular detection of specific resistance determinants.

Perfect concordance between detection of the *mecA* gene and resistance to cefoxitin is biologically consistent with the role of *mecA* in methicillin resistance. In contrast, interpretation involving the *msrA* gene requires caution (Brdová et al. 2024). The *msrA* determinant is associated with resistance to macrolides and streptogramin B but does not confer resistance to lincosamides; therefore, any association between *msrA* and clindamycin non-susceptibility should not be interpreted mechanistically (Kannan and Mankin 2011). Furthermore, the MLS_B induction test (D-test) was not performed, and other determinants of clindamycin resistance were not investigated, which limits mechanistic inferences regarding the macrolide–lincosamide phenotype (Miklasińska-Majdanik 2021).

MRSA prevalence in captive primates varies substantially across facilities, and studies have documented circulation among primates, the environment, and occasionally personnel, highlighting occupational relevance (Hanley et al. 2015; Greenstein 2019; Soge et al. 2016). In our study, methodological and ecological factors may have reduced detection sensitivity, as sampling was performed on rectal mucosa, whereas MRSA screening typically prioritizes nasal, oropharyngeal, and cutaneous sites (Tong et al. 2015; Rossi et al. 2020). Combined with the small sample size and low recovery of *S. aureus*, these factors provide plausible explanations for a negative MRSA finding, even if the pathogen were present at low prevalence (Verhoeven et al. 2012).

Biofilm formation was detectable; however, most isolates were classified as weak producers under the conditions of the microplate assay used (Stepanović et al. 2000). As microplate biofilm assays are sensitive to experimental conditions, comparisons across studies should be made cautiously, and the weak biofilm production observed in this dataset does not support strong claims regarding persistence or clinical impact (Otto 2018). The absence of strong producers and the weak correlations with phenotypic resistance suggest that biofilm formation did not clearly stratify resistance patterns among the evaluated isolates (Schilcher and Horswill 2020; Wu et al. 2024).

Nevertheless, biofilm-associated traits may influence survival on surfaces and in microenvironments, a hypothesis that would require specific study designs, such as environmental sampling and longitudinal follow-up, to be adequately tested (Paharik and Horswill 2016; Otto, 2018; Schilcher and Horswill 2020).

At CETRAS Tangara, fecal material and other organic residues generated during enclosure cleaning are routinely removed and disposed of in a rudimentary on-site composting area, lacking physical barriers or access control, thereby allowing contact with local synanthropic fauna. Although environmental sampling was not conducted, such practices may facilitate environmental dissemination of resistant bacteria and increase opportunities for occupational and ecological exposure, reinforcing the need for improvements in biosafety and waste management protocols (Abosse et al. 2024; Mandal et al. 2025).

This study has limitations, including the sample size and the inclusion of a single rehabilitation center, which may restrict the generalizability of the findings. In addition, whole-genome sequencing or metagenomic approaches (shotgun metagenomics and/or resistome profiling) were not performed; such methods would allow deeper characterization of the resistome and mobilome, evaluation of mobile genetic elements, assessment of isolate relatedness, and more robust inference of AMR transmission routes and potential sources within the rehabilitation environment. Nevertheless, this work provides a baseline, culture-based characterization of viable isolates, using standardized phenotypic testing, MALDI-TOF identification, and targeted detection of key resistance determinants. All isolates were cryopreserved to enable future genomic investigations, and follow-up studies should prioritize whole-genome sequencing and/or metagenomics, expanded sampling of different anatomical niches (nasal/oropharyngeal) and environmental sites, as well as broader molecular panels, to strengthen genotype–phenotype interpretation and One Health risk assessment.

## Conclusion

The investigation achieved its objective by characterizing antimicrobial resistance and biofilm formation in bacteria isolated from rehabilitated *Sapajus libidinosus*. Although the relationship between genotype, phenotype, and biofilm proved limited under the conditions and targets investigated, it was possible to identify isolates with antimicrobial resistance profiles of potential sanitary relevance. Taken together, the findings broaden the knowledge about the ecology of resistance in this context, establish a baseline for microbiological surveillance in fauna screening and rehabilitation, and support the understanding of the microbiological component in rehabilitation and reintroduction scenarios.
