# Correction: Antimicrobial Resistance and Biofilm in Bacteria from Rehabilitated *Sapajus libidinosus*

**DOI:** 10.1007/s10393-026-01809-2

**Published:** 2026-06-01

**Authors:** Denny Parente de Sá Barreto Maia Leite, Gustavo de Oliveira Alves Pinto, Maria Eduarda Uchôa Cavalcanti Moreira da Silva, Valdir Vieira da Silva, Lucilene Martins Trindade Goncalves, Maria Clara Feitosa de Albuquerque, Rafaela Silva Santos, Luana Thamires Rapôso da Silva, Yuri Marinho Valenca, Karolina Rosa Fernandes Beraldo, Maria Aparecida Juliano, Pollyanne Raysa Fernandes de Oliveira, José Givanildo da Silva, Rinaldo Aparecido Mota

**Affiliations:** 1 Graduate Program in Animal Biosciences, Federal Rural University of Pernambuco (UFRPE), Recife, Pernambuco Brazil; 2 Department of Veterinary Medicine, Federal Rural University of Pernambuco (UFRPE), Recife, Pernambuco Brazil; 3 Graduate Program in Veterinary Medicine, Federal Rural University of Pernambuco (UFRPE), Recife, Pernambuco Brazil; 4 Wildlife Screening and Rehabilitation Center (CETRAS-Tangara), Recife, Pernambuco Brazil; 5 Department of Biophysics, Paulista School of Medicine, Institute of Environmental, Chemical and Pharmaceutical Sciences, Federal University of São Paulo (UNIFESP), São Paulo, SP Brazil; 6 Department of Preventive Veterinary Medicine and Animal Production, School of Veterinary Medicine and Animal Science, Federal University of Bahia (UFBA), Salvador, Bahia Brazil

**Correction to: EcoHealth**https://doi.org/10.1007/s10393-026-01777-7.

The original version of this article unfortunately contained error in two author names.

The given name and family name are swapped for the following authors and published incorrectly as follows:

- Denny Parente Sá Barreto Maia de Leite
- Gustavo Oliveira Alves de Pinto

Now, the author names are presented correctly as follows:

- Denny Parente de Sá Barreto Maia Leite
- Gustavo de Oliveira Alves Pinto

The original article has been corrected.

